# Supplementary material for: Evaluation of the Microbial Quality of Hermetia illucens Larvae for Animal Feed and Human Consumption: Study of Different Type of Rearing Substrates
Source: Foods. 2024 May 20;13(10):1587. doi: 10.3390/foods13101587 (PMC11120926; doi:10.3390/foods13101587)
Supplement: Supplementary file 1 [file foods-13-01587-s001.zip › foods-2959391-supplementary/foods-2959391-supplementary-file S2.pdf]

Supplementary Materials S2. Pathogen detection or quantification in *Hermetia illucens* native substrate (A), larvae (B) or frass (C) on various breeding substrates (means log CFU/g ± standard deviation, ND: undetected, (n=): detected with n the number of positive samples)

	Rearing substrate	Organic poultry food	Apple pomace and wheat bran	Bananas, variable, wheat bran	Fruit peel, fruit, vegetable, wheat bran, brewer's grains, chestnut chips	Carrot pomace	Celery pomace	Onion pomace	Wheat Silo	Chicken sandwich	Tuna sandwich	Ham sandwich	Dish prepared Andalusian recipe	Dish prepared Italian recipe	Dish prepared Vendean recipe	Fruit peel, fruit, vegetable, wheat bran, brewer's grains, chestnut chips with 1% beef
**Native substrate**	Number of samples tested	n=3	n=3	n=3	n= 3	n=3	n=3	n=3	n=3	n=1	n=1	n=1	n=1	n=1	n=1	n= 3
	Campylobacter spp.	ND	ND	ND	ND	ND	ND	ND	ND	ND	ND	ND	ND	ND	ND	ND
	Clostridium perfringens presumed	<1.0	<1.0	<1.0	<1.0 (n=2); 3.1	<1.0	<1.0	<1.0	<1.0 (n=2); >4.2	<1.0	<1.0	<1.0	1,0	<1.0	1,0	<1.0
	Coagulase-positive staphylococci (37°C)	<1.0	<1.0	<1.0	<1.0	<1.0	<1.0	<1.0	<1.0	<1.0	<1.0	<1.0	<1.0	<1.0	<1.0	<1.0
	Cronobacter spp.	D (n=1)	ND	ND	ND	ND	ND	ND	D (n=2)	ND	ND	ND	ND	D (n=1)	D (n=1)	ND
	Escherichia coli β-glucuronidase positive	<1.0	<1.0	<1.0	<1.0	<1.0	<1.0	<1.0	2.5 ± 0.2	<1.0	<1.0	<1.0	3.9	<1.60	<1.60	<1.0
	Listeria monocytogenes	ND	ND	ND	ND	ND	ND	ND	ND	ND	ND	ND	ND	ND	ND	ND

	Presumptive *Bacillus cereus* (30°C)	2.2 ± 0.1	2.0 ± 0.8	1.8 ± 0.4; <1.0 (n=1)	3.9 ± 0.0	<1.0	1.6 ± 0.2	<1.0	<1.0 (n=1); 4.0 ± 0.2	<1.0	1.5	<1.0	3,0	1.5	2,0	4.0 ± 0.0
	Salmonella spp.	ND	ND	ND	ND	ND	ND	ND	ND	ND	ND	ND	ND	ND	ND	ND
	Number of samples tested	n=3	n=3	n=3	n= 3	n=3	n=6	n=2	n=2	n=1	n=1	n=1	n=1	n=1	n=1	n= 3
	Campylobacter spp.	ND	ND	ND	ND	ND	ND	ND	ND	ND	ND	ND	ND	ND	ND	ND
	Clostridium perfringes presumed	<1.0	<1.0	<1.0	<1.0	2.2 ± 0.7	<1.0 (n=1); 3.0 ± 1.6; >4.2 (n=3)	<1.0; >4.2	3.8; >4.2	<1.0	<1.0	<1.0	<1.0	<1.0	<1.0	<1.0
**Larvae**	Coagulase-positive staphylococci	<2.0	<2.0	<2.0 (n=1); <1	<1.0	<1.0	<1.0	<1.0	<1.0	<1.0	<1.0	<1.0	<1.0	<1.0	<1.0	<1.0
	Cronobacter spp.	ND	ND	ND	ND	D (n=1)	D (n=2)	ND	ND	ND	ND	ND	ND	ND	ND	ND
	Escherichia coli β-glucuronidase positive	>3.1	3.7 ± 0.1	1.9±1.2; <1.0 (n=1)	3.0 ± 1.5	<1 (n=1); 2.1 ± 0.9	<1.6 (n=1); 3.5 ± 0.8	3.2; >6.2	5.2; >6.2	2.5	3.3	1	3.1	<1.0	2.3	4.4 ± 0.0
	Listeria monocytogenes	ND	ND	ND	ND	ND	ND	ND	ND	ND	ND	ND	ND	ND	ND	ND
	Presumptive *Bacillus cereus*	<1.0	<1.0	<1.0	<1.0	<1.0	<1.0	<1.0	<1.0	<1.0	<1.0	<1.0	<1.0	<1.0	<1.0	<1.0
	Salmonella spp.	ND	ND	ND	ND	ND	ND	ND	ND	ND	ND	ND	ND	ND	ND	ND
**Frass**	Number of samples tested	n=3	n=3	n=4	n= 3	n=3	n=6	n=2	n=3	n=1	n=1	n=1	n=1	n=1	n=1	n= 3
	Campylobacter spp.	ND	ND	ND	ND	ND	ND	ND	ND	ND	ND	ND	ND	ND	ND	ND

Clostridium perfringes presumed	<1.0	<1.0	1.9; <1.0 (n=3)	<1.0	<1.0	<1.0	<1.0	<1.0 (n=2); >4.2	<1.0	<1.0	<1.0	<1.0	<1.0	<1.0	<1.0
Coagulase-positive staphylococci (37°C)	<1.0	<1.0	<1.0	<1.0	<1.0	<1.0	<1.0	<1.0	<1.0	<1.0	<1.0	<1.0	<1.0	<1.0	<1.0
Cronobacter spp.	ND	ND	ND	ND	ND	D (n=3)	D (n=1)	D (n=2)	ND	ND	ND	ND	ND	ND	ND
Escherichia coli β-glucuronidase positive	<1.0 (n=1); 3.2 ± 1.2	>6.2 (n=2); 6.0	<1.0 (n=1); 2.8 ± 1.0	3.8 ± 0.8	<1.0	<1.0 (n=3); 4.2 ± 1.6	>6.2; 4.9	5.7 ± 0.2	4,0	<1.0	<1.0	3.2	3.4	4.1	4.0 ± 0.5
Listeria monocytogenes	ND	ND	ND	ND	ND	ND	ND	ND	ND	ND	ND	ND	ND	ND	ND
Presumptive *Bacillus cereus* (30°C)	<1.0; 2.3; >4.2	>4.2 (n=1); 2.3 ± 0.3	2.2 ± 0.6	<1.0 (n=1); 3.4 (n=1)	<1.0; 1.9 ± 0.4	<1.0 (n=3); 2.4 ± 0.3	<1.0 ; >4.2	3.9 ± 0.1	<1.0	<1.0	<1.0	>4.2	<1.0	<1.0	<1.0 ; 3.5 ± 0.1
Salmonella spp.	ND	ND	ND	ND	ND	ND	ND	D (n=1)	ND	ND	ND	ND	ND	ND	ND
